# Comparative Fecal Metabolomes of Silkworms Being Fed Mulberry Leaf and Artificial Diet

**DOI:** 10.3390/insects11120851

**Published:** 2020-11-30

**Authors:** DaoYuan Qin, GenHong Wang, ZhaoMing Dong, QingYou Xia, Ping Zhao

**Affiliations:** 1 State Key Laboratory of Silkworm Genome Biology, Southwest University, Chongqing 400715, China; qdyddinsist@email.swu.edu.cn (D.Q.); wanggh168@swu.edu.cn (G.W.); dongzhaoming@swu.edu.cn (Z.D.); xiaqy@swu.edu.cn (Q.X.); 2 Biological Science Research Center, Southwest University, Chongqing 400715, China; 3 Chongqing Key Laboratory of Sericultural Science, Chongqing Engineering and Technology Research Center for Novel Silk Materials, Southwest University, Chongqing 400715, China

**Keywords:** artificial diet, *Bombyx mori*, feces, metabolic profiling, mulberry leaf

## Abstract

**Simple Summary:**

Silkworm (*Bombyx mori*) is an oligophagous insect and their diets have preference for mulberry leaves. However, mulberry leaves are seasonal resources, not available in the winter, which severely limits the silkworm rearing and cocoon production. The aim of this study was to investigate the mechanism that caused the stunted growth and the low efficiency of silk protein synthesis of silkworms fed with artificial diet by analyzing the fecal metabolome. Compared to silkworm reared with mulberry leaves, the contents of the amino acids, carbohydrates and lipids in the feces of silkworm fed with artificial diet obviously decrease, while some organic acids, such as urea and citric acid significantly increase. These findings provide new insights for the improvement of silkworm artificial feed through metabolomics research.

**Abstract:**

Metabonomics accurately monitors the precise metabolic responses to various dietary patterns. Metabolic profiling allows simultaneous measurement of various fecal metabolites whose concentrations may be affected by food intake. In this study, we analyzed the fecal metabolomes of silkworm (*Bombyx mori*) larvae reared on fresh mulberry leaves and artificial diets. 57 differentially expressed metabolites were identified by gas chromatography–mass spectrometry. Of these, 39 were up-regulated and 18 were downregulated in the mulberry leaf meal group. Most of the amino acids, carbohydrates and lipids associated with physical development and silk protein biosynthesis were enriched in silkworms reared on mulberry leaves. In contrast, the urea, citric acid, *D*-pinitol, *D*-(+)-cellobiose and *N*-acetyl glucosamine levels were relatively higher in the silkworm feeding on the artificial diets. The findings of this study help clarify the association between diet and metabolic profiling.

## 1. Introduction

Silkworm (*Bombyx mori*) is one of the most economically important insects worldwide. It has co-evolved with mulberry. The silkworm is an oligophagous insect, whose exclusive food is mulberry leaves. Silkworms usually make the best quality cocoons in spring, because their food, mulberry leaves, are the best quality in this season. In contrast, the production performance of silkworms reared in autumn is usually lower than that in spring, which was mainly due to mulberry leaves with poor quality [1,2,3]. This feeding characteristic of the silkworm greatly limits the development of the sericulture. In rural regions of China, sericulturists tried to supplement feeding silkworm larvae with flour or sugar when the mulberry leaf quality was poor. Previous researchers also attempted to improve silkworm growth by spraying nutrients on the mulberry leaves [4,5]. With the development of sericulture [6], people are eager to develop artificial diet for silkworm. On the one hand, silkworm could be reared throughout the year; on the other hand, it is expected to meet the demand for producing silk [7,8,9,10] with various characteristics by using artificial diet. In recent decades, scientists have made considerable progress in the development of artificial diet for silkworms but it is still far from being applied to sericulture.

The mulberry (*Morus* sp.) leaf nutrients profile served as the gold standard food for artificial diet formulation. Mulberry leaves contain sugars, lipids, proteins, minerals, vitamins [11] and flavonoids [12]. There are numerous practical challenges in the design of optimal silkworm diets. Nutritional requirements differ depending on whether the silkworms produce cocoon [13] or eggs. In the classical diet formulation approach, the intake levels of each of the numerous macro- and micronutrients required by the silkworm were painstakingly determined [14,15,16,17,18,19]. This single-variable strategy has been the foundation of nutrition science.

Metabonomics measures the real physiological regulatory process endpoints of the molecular interactions among the host, its gut microbiota, environmental factors and diet [20,21]. Metabonomics is implemented to improve artificial diets and reduce chronic disease burdens. The injection of glycine into silkworm hemolymph can increase silk protein biosynthesis [22]. Metabonomics studies on silkworm hemolymph have demonstrated that rearing silkworms on artificial diets instead of fresh mulberry induced vitamin deficiencies and disorders of amino acid and uric acid metabolism [23]. Megastigmane Sesquiterpenes, which showed promotion activity on HO-1 and SIRT1, was identified in the excrement of silkworm reared with mulberry leaves by gas chromatography-mass spectrometry (GC-MS) [24].

In this study, we used GC-MS to analyze and compare the fecal metabolome of silkworms maintained on artificial diets with that of silkworms fed mulberry leaves. The findings of this study reveal the metabolic differences among silkworms raised on various diets and provide valuable data for the improvement of artificial diet.

## 2. Materials and Methods

### 2.1. Experimental Insects

**Figure 1:**
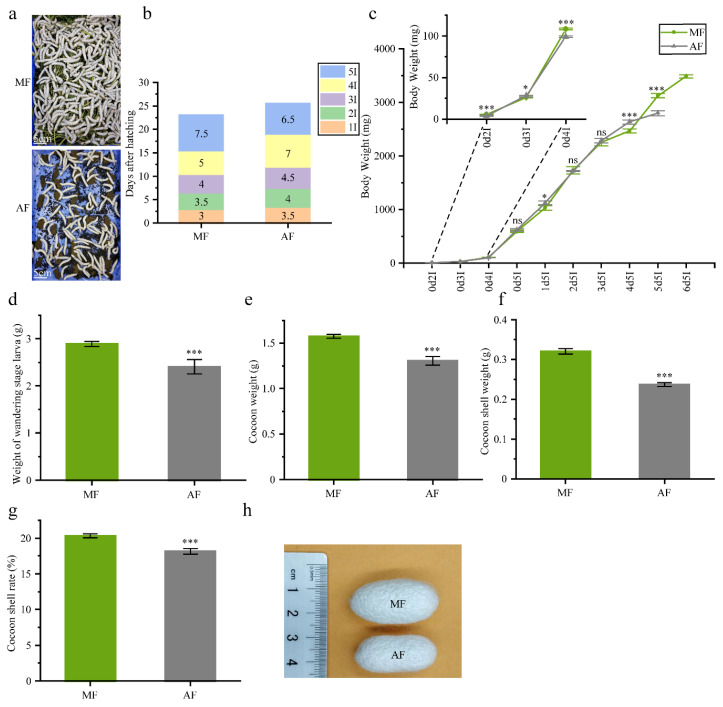
Comparison of the effects of fresh mulberry leaves and artificial diets on silkworm development and cocoon yield. (**a**) 120-h, 5th instar silkworm larvae feeding on mulberry leaf and artificial diet in plastic casing. Bar = 5 cm (**b**) Development time at each silkworm instar. 5I, 4I, 3I, 2I and 1I represent 5th, 4th, 3rd, 2nd and 1st instars, respectively. (**c**) Changes in average body weight of silkworms fed fresh mulberry leaf and artificial diet (*n* = 100 individuals/group, three groups). Stage labels: the first number shows day and the second number indicates instar (e.g., 0d5I means day 0 in the 5th instar). (**d**) weight of wandering-stage larvae (*n* = 100 individuals/group, three groups). (**e**) Cocoon weight (*n* = 30 individuals/group, three groups). (**f**) Cocoon shell weight (*n* = 30 individuals/group, three groups). (**g**) Cocoon shell rate (*n* = 30 individuals/group, three groups). (**h**) Cocoons from mulberry leaf and artificial diets groups. MF and AF represent larvae reared on fresh mulberry leaf and artificial diet, respectively. Data were expressed as mean ± SEM. Differences of data was assessed by Student’s *t*-test (Two tailed, two sample equal variance hypothesis), * *p* < 0.05; *** *p* < 0.001; ns *p* > 0.05.

Silkworm strain Liangguang II was obtained from our laboratory at Southwest University, Beibei, China. Individuals were reared either on artificial diets (A) or fresh mulberry leaves (M). Each diet was done in three group containing about 150 larvae, thus in three replicates. The mulberry leaves were from the Chinese ‘JiaLing20’ variety cultivated in a field at Southwest University. The mulberry trees are about 10 years old. Fresh mulberry leaves were picked from mulberry trees every day. Silkworms were fed with enough fresh mulberry leaves twice one day. Bed cleaning was performed daily in every instars stages in MF. In AF group, Silkworm was also fed with enough artificial diets and bed cleaning was performed once during the first, second and third instar stages at the end of the moulting period, twice in the fourth instar stage and daily in the fifth instar stage. Silkworms reared with either mulberry leaves or artificial diet were kept at 25 °C and under a 12 h light/12 h dark photoperiod [13]. Figure 1a shows silkworm larvae reared on mulberry leaf and artificial diet on the 5th day of 5th-instar.

### 2.2. Experimental Artificial Diet

The artificial diet composition (*w*/*w*) was as follows: 30% defatted soybean powder, 25% mulberry leaf powder, 19.6% starch, 10% cellulose powder, 8% agar, 2% vitamin C, 2% citric acid, 1.5% vitamin B complex, 1% mineral salts complex, 0.5% Potassium Sorbate, 0.2% choline chloride and 0.2% Calcium propionate. Add 3 times the amount of water (ratio by weight) to the unprocessed artificial diets and heat it at 100 °C for 35 min. After processing, refrigerate it by film and finish it up within a week.

### 2.3. Fecals Samples Collection

The feces excreted within 1 h was collected on the 5th day of 5th-instar after silkworms fed with either mulberry leaves or artificial diet. The samples were collected in six replications for each group, quick-frozen in liquid nitrogen and stored at −80 °C.

### 2.4. Measurement of Developmental Parameters

The statistics of cocoon production included cocoon weight, cocoon shell weight and cocoon shell ratio (%). Cocoon weight (*n* = 30), cocoon shell weight (*n* = 30) and cocoon shell ratio (%) [(cocoon shell weight/cocoon weight) × 100] were recorded on the sixth day of the pupal stage. The fresh body weight of the newly ecdysized larvae (*n* = 100) were measured in the same developmental stage in the 2nd, 3rd, 4th and 5th instar. The fresh body weight of the larvae (*n* = 100) was measured every day of the 5th instar stage. Data were expressed as mean ± SEM. Differences of data was assessed by Student’s *t*-test (Two tailed, two sample equal variance hypothesis). The data for Figure 1c–g was provided in Source Data. When the newly ecdysized larvae rate (%) [(newly ecdysized larvae/total larvae) × 100] reaches 90%, we define it as entering the next instar.

### 2.5. Sample Processing for GC-MS

Three biological replicates were analyzed with GC-MS for both artificial diet (A) and mulberry leaf (M). For fecal samples, 6 biological replicates were used for both MF and AF. Freeze-dried fecal, fresh mulberry leaves (using the sixth leaf from the top of the mulberry branch) and cooked artificial diet samples were crushed with a Micro Smash (MS-100R, TOMY) with a zirconia bead (ZB-30, from TOMY Digital Biology, Fukuoka, Japan) for 1.5 min at 3000 rpm and 4 °C. Then 100 mg powder was weighed out and extracted overnight at 4 °C in either 1 mL pure methanol or 1 mL of 70% (*v*/*v*) aqueous methanol to isolate lipid soluble or water soluble metabolites, respectively. Following centrifugation at 14,000× *g* and 4 °C for 15 min, 0.6 mL supernatant of each extract was mixed, vortexed and passed through a 0.22 μm filter. Four hundred microliters were dried in a refrigerated CentriVap Concentrator (LABCONCO, Kansas City, MO, USA) overnight. Extraction was performed according to a previously published method [25].

Eight microliters of methoxyamine solution (20 mg mL^−1^ in pyridine) was used to dissolve the dry metabolites. The solution was immersed in a water bath at 37 °C for 1.5 h for the oximation followed by 15 min of ultrasound. Then 60 μL of N-methyl-N-(trimethylsilyl) trifluoroacetamide was added for the sialylation reaction at 37 °C for 1 h. Then 10 μL of n-heptane was added to terminate the reaction. After 20 min centrifugation at 10 °C and 14,000 rpm, the supernatant was transferred for the subsequent GC-MS analysis according to previously published methods [26,27].

### 2.6. Instrument Parameters for Data Acquisition

Fecal or diet samples extracts (1 μL) with split ratio = 10:1 were analyzed in an Agilent 7890B-5977A system fitted with a DB-5MS column (0.25 μm × 0.25 mm × 30 m; Agilent Technologies Inc., Santa Clara, CA, USA). The injection temperature was 300 °C, the transfer interface was set to 280 °C and the ion source was adjusted to 230 °C. The oven temperature program consisted of an initial 70 °C for 3 min followed by an increase to 170 °C at 5 °C min^−1^, 234 °C at 4 °C min^−1^, 270 at 5 °C min^−1^, ramping up to 300 °C at 10 °C min^−1^ and holding for 5 min. The detector voltage was set to 0.93 kV. The metabolite EI ionization voltage was set to 70 eV. Full scan mode (m/z: 33−600) was used to acquire the mass signals.

### 2.7. Metabolomics Data Preprocessing and Analysis

Raw GC-MS mass spectra were converted to AIA data format files and processed using XCMS in R software (https://xcmsonline.scripps.edu/index.php). Metabolites were identified by comparing mass spectra and retention indices with those in the National Institute of Standards and Technology (NIST, Gaithersburg, MD, USA) library and match values over >700 were used. Orthogonal partial least squares discriminant analysis (OPLS-DA) was performed in SIMCA-P v. 14.0 to identify differential metabolites abundance in MF compared with AF. All the experiment for MF and AF were done in 6 biological replicates. Student’s *t*-test (Two tailed, Two-sample equal variance hypothesis) was used to analyze the changes in the metabolites abundance between the MF and AF by using Microsoft Excel 2016 (Microsoft Corp., Redmond, WA, USA). We used the abundance values of metabolites for OPLS-DA. We obtained the KEGG accession numbers of the identified metabolites (http://www.kegg.jp/) and mapped the metabolic pathways based on the KEGG database. The structures of the compounds were obtained from ChemSpider (https://www.chems-pider.com/) as Mol file and the Mol file was opened with KingDraw (http://www.kingdraw.cn/) software. The heatmap function of the R language package was used for the heat map analysis (www.r-project.org). SPSS software was used to analyze the correlation of metabolites (Table S3).

## 3. Results

### 3.1. Chemical Components Analysis of Mulberry Leaf and Artificial Diet

One hundred and twenty-nine chemical components were annotated from the detected spectral features from GC-MS, using the available database (NIST, Gaithersburg, MD, USA). These chemical components including a large variety of substances, such as carbohydrates, amino acids, organic acids and fatty acids (Table S2). Among them, there are 28 chemical components with match value greater than 700, which showed significant difference between mulberry leaf (M) and artificial diet (A). The top 8 chemical components showing significantly higher level in M than that in A were 9,12,15-octadecatrienoic acid, glyceryl-glycoside TMS ether, sucrose, tyrosine, Xylitol, *D*-(+)-trehalose, glycoside and *myo*-inositol. The top 8 chemical components showing significantly higher level in A than that in M were citric acid, *D*-(−)-tagatose, sorbic acid, glutamic acid, serine, *D*-pinitol, *L*-isoleucine and *D*-(+)-xylose.

### 3.2. Impact of Artificial Diet on Silkworm Developmental Factors and Cocoon Production

The feeding period for the silkworms reared on the artificial diet was 25.5 d, which was 10.9% longer than that for the silkworms raised on mulberry leaf (~23 d). However, the 5th instar was 1 d shorter in the former than in the latter group. The length of the 1st, 2nd and 3rd instar were 3.5 d, 4 d and 4.5 d long, respectively, on artificial diets meal group (AF). Each of these stages was 12 h shorter in the mulberry leaf group (MF). A similar trend was observed for the 4th instar (Figure 1b).

Different diets had different effects on larval body weight increase. For AF, the larval body weight of the 5th instar, reached a maximum of 2.79 g by day 5 (120 h feeding). For MF, however, the 5th instar reached a maximum body weight of 3.48 g by day 6 (144 h feeding) (Figure 1c). The average weight of mature silkworms feeding on mulberry leaf and the artificial diet were 2.89 g and 2.41 g, respectively (Figure 1d).

We also measured and compared the cocoon, cocoon shell and the cocoon shell rates of both groups. The cocoon weights for MF were heavier than those for AF. The cocoon shell weights were 0.32 g and 0.24 g for MF and AF, respectively. The cocoon shell ratio for MF was 20.32%, which was 12% higher than that for AF (18.13%). Hence, the mulberry leaf diet resulted in relatively higher cocoon production (Figure 1e–g) and the cocoons in AF were smaller than those in MF (Figure 1h).

### 3.3. Fecal Extract Metabolic Profiles of Silkworms Reared on Fresh Mulberry Leaf and Artificial Diet

**Figure 2:**
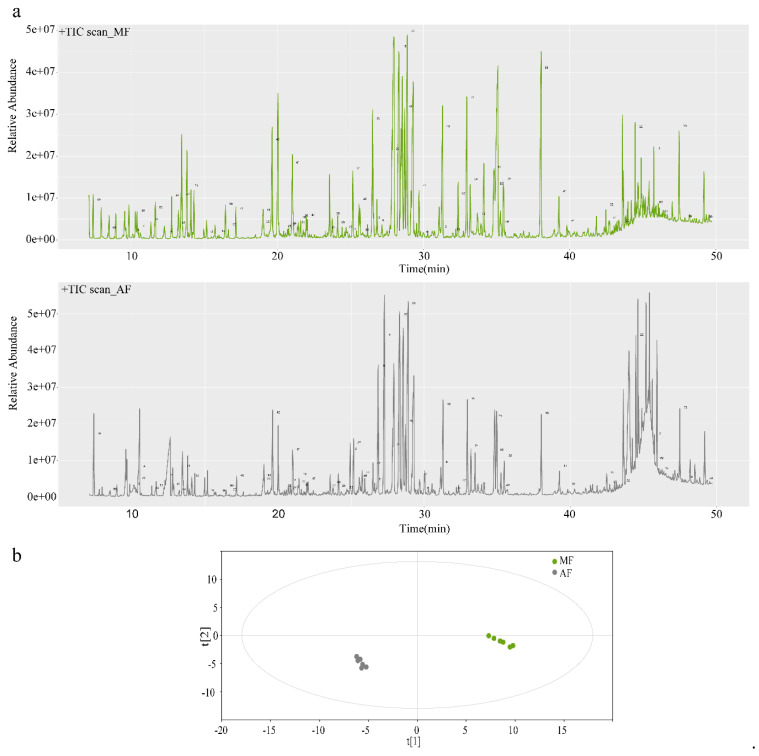
Relative gas chromatography-mass spectrometry (GC-MS) base peak intensity ion chromatograms (TICs) (**a**) and orthogonal partial least squares discriminant analysis (OPLS-DA) for feces of mulberry leaf group (MF) and artificial diet group (AF) (**b**). MF and AF are the fecal samples from larvae reared on fresh mulberry leaf and artificial diet, respectively. MF, *n* = 6 replicates; AF, *n* = 6 replicates.

Figure 2a shows GC-MS base peak intensity chromatograms for AF and MF feces. Visual examination and comparison of the GC-MS chromatograms revealed obvious differences between the AF and MF fecal samples (Figure 2a). The peak number of each compound which was listed in Table S1 was identified in chromatograms. OPLS-DA disclosed good separation between groups based on the fecal samples (Figure 2b).

### 3.4. Diets-Related Fecal Metabolite Analysis

Table 1 lists the fecal metabolites whose levels significantly differed between MF and AF (*p* < 0.05). A heat-map based on the abundance of the identified metabolites is shown in Figure S1. Fifty-seven different metabolites were identified. Of these, 39 were upregulated and 18 were downregulated in MF relative to AF. The top 10 metabolites showing significantly higher level in MF than that in AF were *L*-methionine, *D*-allose, *L*-alanine, 9,12,15-octadecatrienoic acid, 2-keto-L-gluconic acid, benzoic acid, *L*-leucine, *L*-valine, galactinol and α-linolenic acid. Hence, most of the MF metabolites were amino acids and lipids. The top 10 metabolites showing significantly higher level in AF than that in MF were *D*-pinitol, 3-hydroxyanthranilic acid, *D*-(+)-cellobiose, sorbic acid, butanoic acid, *N*-acetyl glucosamine, urea, citric acid, *D*-gluconic acid and propanoic acid. 

### 3.5. Fecal Amino Acid, Lipid and Sugar Analyses

**Figure 3:**
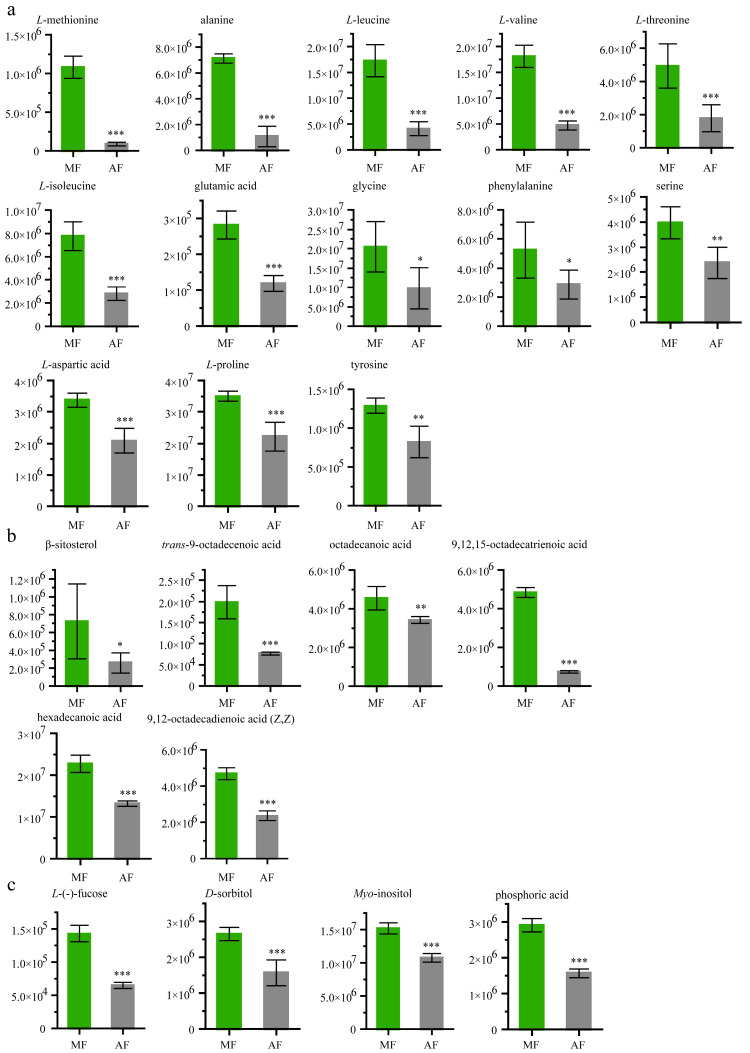
Abundance analysis of 13 amino acids and six lipids identified in feces from different diets. (**a**) Main amino acids, (**b**) main lipids, and (**c**) sugars and inorganic acid. Data were expressed as mean ± SEM. Differences of data was assessed by Student’s *t*-test (Two tailed, Two-sample equal variance hypothesis), * *p* < 0.05; ** *p* < 0.01; *** *p* < 0.001; ns, *p* > 0.05. MF and AF are larvae reared on fresh mulberry leaf and artificial diet, respectively. MF, *n* = 6 replicates; AF, *n* = 6 replicates.

Figure 3a shows that levels of some amino acids were higher in MF than in AF. The *L*-methionine abundance in the mulberry leaf group was 12.87 fold higher than that in the artificial diet group. The tyrosine level was 1.57-fold higher in MF than in AF. The levels of *L*-alanine, *L*-leucine, *L*-valine, *L*-threonine, *L*-isoleucine, glutamic acid, glycine, phenylalanine, serine, *L*-aspartic acid and *L*-proline, were 6.56-fold, 4.22-fold, 3.82-fold, 2.8-fold, 2.78-fold, 2.37-fold, 2.1-fold, 1.82-fold, 1.67-fold, 1.62-fold and 1.58-fold higher, respectively, than they were in MF. The chemical structures of these compounds are shown in Figure S2.

The abundances of six different lipids (9,12,15-octadecatrienoic acid, β-sitosterol, *trans*-9-octadecenoic acid, 9,12-octadecadienoic acid (Z,Z), hexadecenoic acid, octadecanoic acid) were greater by 6.51-fold, 2.78-fold, 2.56-fold, 1.99-fold, 1.72-fold and 1.32-fold, respectively, in the MF feces than in the AF feces. (Figure 3b). The levels of *L*-(−)-fucose (*t* value: −14.04; degree freedom: 6; *p* value: 8 × 10^−6^)*, D*-sorbitol (*t* value: −6.63; degree freedom: 7; *p* value: 0.0003), *myo*-inositol (*t* value: −10.44; degree freedom: 10; *p* value: 1 × 10^−6^) and phosphoric acid (t value: −14.62; degree freedom: 9; *p* value: 1 × 10^−7^), were >2.19-fold, >1.69-fold, >1.4-fold, >1.85-fold higher in MF, respectively, than they were in AF (Figure 3c).

### 3.6. Fecal Organic Acid and Carbohydrate Analyses

**Figure 4:**
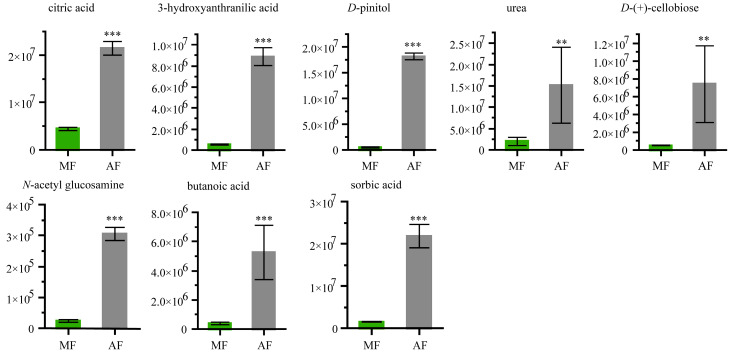
Relative abundances of sugars, organic acids and urea in feces from different diets. Data were expressed as mean ± SEM. Differences of data in silkworm subjects was assessed by Student’s *t*-test (Two tailed, Two-sample equal variance hypothesis), ** *p* < 0.01; *** *p* < 0.001; ns, *p* > 0.05. MF and AF are larvae reared on fresh mulberry leaf and artificial diet, respectively. MF, *n* = 6 replicates; AF, *n* = 6 replicates.

Figure 4 shows the fecal metabolites that were more abundant in AF than in MF. Citric acid, urea, *N*-acetyl glucosamine, butanoic acid, sorbic acid, *D*-(+)-cellobiose, 3-hydroxyanthranilic acid and *D*-pinitol, were 4.8-fold, 7.7-fold, 13.12-fold, 13.56-fold, 14.81-fold, 15.15-fold, 17.69-fold and 37.28-fold higher, respectively, in AF than in MF (Figure 4). The chemical structures of these compounds are shown in Figure S3.

### 3.7. Pathway for the Metabolites with Different Abundance in Fecal

**Figure 5:**
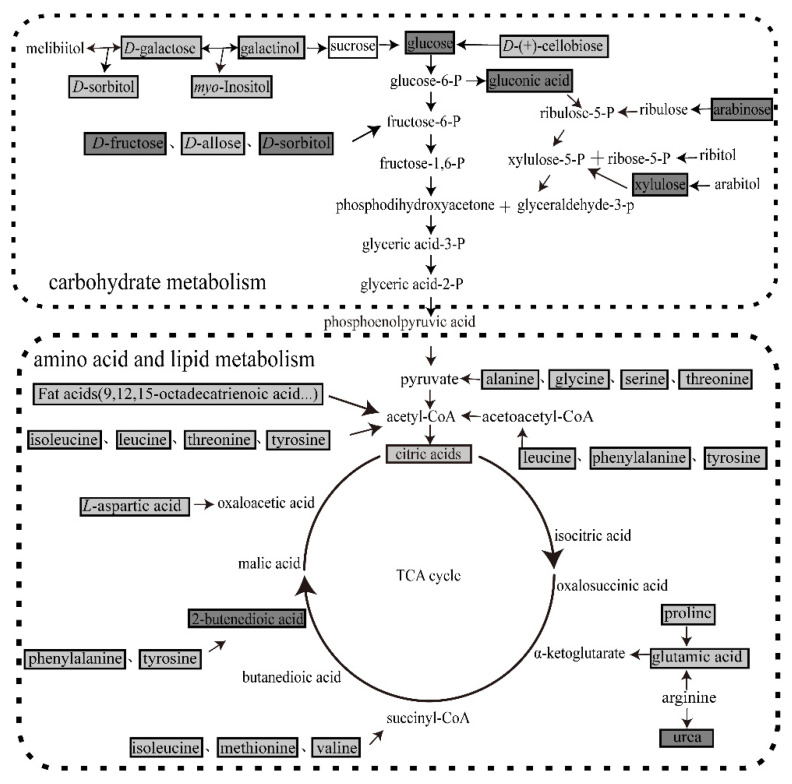
Metabolic pathways involved in silkworm fecal metabolite production. Metabolic pathways were mapped according to KEGG (http://www.kegg.jp/). Light grey boxes indicate that the abundance of fecal silkworm metabolites in the mulberry leaf group were significantly higher than those in the artificial diet group. Dark gray boxes reveal that the abundances of the fecal silkworm metabolites in the mulberry leaf group were significantly lower than those in the artificial diet group. Black font with black frame represents metabolites whose levels did not significantly differ between treatments. Black font indicates metabolites not detected in this experiment.

A total of 13 amino acids, 5 fatty acids, 13 sugars and 1 organic acid showing different abundance between AF and MF were mapped into an integrated metabolic pathway (Figure 5). All amino acids could be catabolized to common tricarboxylic acid cycle (TCA) intermediates such as α-ketoglutarate, succinyl-CoA, 2-butenedioic acid, acetyl-CoA and oxaloacetic acid (Figure 5). *D*-(+)-cellobiose, sucrose, galactinol and *D*-galactose could be degraded into glucose (Figure 5). *D*-fructose, *D*-allose, *D*-sorbitol could be transformed into fructose-6-phosphate (Figure 5). The aforementioned carbohydrates would then feed into the glycolytic pathway. Fatty acids such as 9,12,15-octadecatrienoic acid were oxidized to acetyl-CoA which feeds into the TCA cycle (Figure 5). Hence, the TCA cycle pathway is severely affected in silkworms fed artificial diet.

## 4. Discussion

### 4.1. Certain Metabolites Were More Abundant in The Feces of the Artificial Diet Group than They Were in the Feces of the Mulberry Leaf Group

In animals, urea or uric acid are the final catabolites of nitrogenous substances such as proteins and amino acids. An imbalance in the dietary amino acid composition leads to poor growth and an increase in uric acid excretion [28]. In the present study, the uric acid level (Table S1) was higher in the AF feces than in the MF feces, although the difference was not significant. The urea concentration was significantly higher in the AF feces (7.7-fold) than in the MF feces. Urea produced in the silkworm midgut can be hydrolyzed to ammonia by the urease in mulberry leaf [29]. Nevertheless, urease is deactivated after the mulberry leaf is processed into artificial diets. Urease gene overexpression in the silkworm midgut can lower urea concentrations in the midguts and hemolymphs of silkworm larvae fed artificial diets [30].

Citric acid is a TCA intermediate. Here, it was comparatively more abundant in the AF feces than in the MF feces. The TCA cycle links the carbohydrate, lipid and amino acid metabolic pathways [31]. In the TCA cycle, citrate synthase catalyzes the conversion of acetyl-CoA and oxaloacetate to citric acid and the latter is converted by aconitase to cis-aconitate [31]. The TCA cycle furnishes the cells with a steady ATP supply [31]. The use of citric acid as a feed additive has been widely reported in recent years. Dietary citric acid is anti-inflammatory in the intestine. It alleviated soybean meal-induced intestinal diseases in turbot [32]. However, it also inhibits several key glycolytic enzymes such as phosphofructokinase 1 (PFK1) and 6-phosphofructo-2-kinase/fructose-2,6-biphosphatases (PFK2) [33]. It is unknown whether high dietary citric acid intake promotes silkworm growth. *D*-pinitol was detected in soybean [34] which has often been added to artificial diets as the main protein source [23,28,35]. However, soybean contain substances that inhibit feeding in silkworm larvae [36]. The addition of pinitol to artificial diets inhibited growth and feeding in the larvae of *Heliothis zea* [37,38]. In the present study, we found that the *D*-pinitol content was 37-fold higher in AF feces than that in the MF feces. Hence, we speculated that silkworm larval growth might be enhanced by reducing the amount of *D*-pinitol in artificial diets. Since the artificial diet of insects is rich in nutrients and provides an ideal environment for the growth of molds, antifungal additives are usually used to reduce dietary contamination. Sorbic acid is generally used as an additive to inhibit the growth of molds and is widely used in insect feed [28,39]. However, sorbic acid has been found to have negative impact on rice leaf folder (*Cnaphalocrocis medinalis*) larvae [40]. The sorbic acid level was 14.81-fold higher in AF feces than in MF feces. The use of high level of sorbic acid might be another disadvantage of artificial diet. Butanoic acid is generally produced by the metabolism of the bacteria in the intestine [41]. Some researchers have shown that feeding mice with engineered *Bacillus subtilis* SCK6, which produces butanoic acid, can cause reduced food intake and weight loss in mice [42]. And we found that the butanoic acid content in AF feces was 13.56 times higher than that of the MF feces. The intestinal microbial community diversity of silkworm larvae fed with artificial diet was lower than that of silkworm larvae fed with mulberry leaves [43]. It would be interesting to further explore which gut microbiota causes the intestinal butanoic acid produced after silkworms reared with artificial diets, which further affected the growth and development of the artificial diets-fed silkworm. The *N*-acetyl glucosamine level was 13.12-fold higher in AF feces than that in in MF feces. In adult *Aedes aegypti*, chitin is an important part of the perifeeding matrix and it is synthesized de novo in response to diet [44]. *N*-acetyl glucosamine was usually from the hydrolyzation of chitin in insects [45]. Foliage-fed larvae (*Heliothis virescens*) had a significantly thicker peritrophic matrix than diet-fed larvae [46]. It is inferred that the chitin degradation pathway of the perifeeding matrix of the AF is more active than that in MF, which might lead to the metabolic disorder of *N*-acetyl glucosamine in the AF. The 3-hydroxyanthranilic acid level was 17.6-fold higher in AF feces than in MF feces. Injection of 3-hydroxyanthranilic acid into adult flies caused short-term wing muscle motor dysfunction. This substance was also toxic to *Locusta migratoria* neurosecretory cells [47]. High 3-hydroxyanthranilic acid levels might also disrupt silkworm larva nervous and motor systems. Therefore, reducing the amount of 3-hydroxyanthranilic acid might be helpful for optimizing artificial diet for silkworm. The cellobiose level was 15-fold higher in the AF feces than in the MF feces. Cellulose occurs in both mulberry leaf and artificial diets [48]. The cellulolytic bacteria in the midguts of silkworm larvae ingesting mulberry leaf can convert cellulose to cellobiose [49]. Silkworm larva midguts produce β-glucosidase which can hydrolyze cellobiose to glucose [50]. Several enzymes convert cellobiose to glucose in the guts of *Globitermes brachycerastes* [51]. Diversity of the intestinal microbial community was lower in silkworm larvae reared on artificial feed than that in silkworm larvae fed mulberry leaf [43]. We speculated that cellobiose metabolism was perturbed when the silkworm larvae consumed the artificial diet.

### 4.2. Certain Metabolites Were Less Abundant in the Feces of Silkworm Larvae Given the Artificial Diet than They Were in the Feces of Silkworm Larvae Ingesting Mulberry Leaf

Amino acids are both protein components and signaling molecules. They regulate food intake, gene expression, protein phosphorylation and intercellular communication [52]. Glycine, serine, alanine and tyrosine are the main amino acid constituents of silk [53]. Reduction in the levels of these four amino acids in AF may interfere with silk protein biosynthesis in silkworm larvae raised on this diet. Another study reported that *L*-glutamic, *L*-alanine and *L*-aspartic acid sufficed to promote food consumption in Drosophila in a dose-dependent manner [52]. Hence, the addition of amino acids to artificial diets might improve silkworm larva feeding performance.

Mulberry leaf contains myristic, palmitoleic, oleic, 9,12,15-octadecatrienoic, 9,12-octadecadie-noic (Z,Z), hexadecenoic and octadecanoic acids [11]. The levels of 9,12,15-octadecatrienoic, *trans*-9-Octadecenoic, 9,12-octadecadienoic (Z,Z), hexadecenoic and octadecanoic acids were higher in the MF feces than that in the AF feces. The β-sitosterol level was 2.78-fold higher in the MF feces than that in the AF feces (Figure 4). In insects, sterols are structural cell membrane components and ecdysone precursors. Ecdysone determines individual growth time by inducing ecdysis and metamorphosis [54]. All insects require dietary sterols as they cannot biosynthesize them. β-sitosterol supplementation is highly beneficial to silkworm larvae [55]. Administration of the appropriate species and concentrations of phytosterols is vital to silkworm larva growth and development.

Sugar is a carbon and energy source for silkworm larvae [56]. It also promotes their feeding. *D*-sorbitol somewhat stimulated silkworm larva feeding [16]. Behavioral experiments showed that *myo*-inositol plus sucrose increased silkworm larva feeding activity [57]. The abundance of *D*-sorbitol and *myo*-inositol in the AF was significantly lower than that in the MF. Relative lower level of these three substances might be one of the reasons for poor growth and development of silkworms in the AF group.

The present study demonstrated that the MF feces contained 1.85-fold more phosphoric acid than AF feces. Phosphorus is an essential mineral and a basic cell component. All nucleic acids contain phosphorus. This element is also a constituent of ATP which provides cellular energy [58]. Silkworm larvae acquire their phosphorus from their diet. The hemolymph phosphate level was increased in late 5th instar larva ingesting mulberry leaf [59]. Potassium phosphate supplementation in artificial diets comprising phosphorus-deficient mulberry leaf powder could recover silkworm larva growth [60]. *Manduca sexta* caterpillars maintained on standard and high-phosphorus artificial diet were significantly heavier than those fed a low-phosphorus artificial diet [61]. Future research should explore the influences of phosphorus supplementation in artificial diets on silkworm larvae.

Figure 5 shows TCA pathway is severely affected in silkworms fed artificial diet. Further studies are required to clarify the effects of these metabolites showing different abundance in this pathway on silkworm larva growth and development. Future research should also examine the individual effect of differentially expressed metabolites in silkworm larvae maintained on MF and AF.

## 5. Conclusions

In this study, the metabonomics method was used to compare the differences in the metabolites in the feces of silkworms reared with mulberry leaf and artificial diet. The results showed that, compared with the artificial diet group, 39 metabolites were up-regulated and 18 metabolites were down regulated in mulberry leaf group. These metabolomics data will provide information for the improvement of artificial diet, which will help us better understand the relationship between silkworm’s diet and growth and provide a reference for the subsequent development of artificial diets for silkworms or other insects.

## Figures and Tables

**Table 1:** Differential abundance metabolites between mulberry leaf and artificial diet groups.

Metabolite	Category	P	AF/MF
*L*-methionine	Amino acid	1 × 10^−8^	0.08
*D*-allose	Sugar	4 × 10^−10^	0.15
*L*-alanine	Amino acid	9 × 10^−9^	0.15
9,12,15-octadecatrienoic acid	Fatty acid	4 × 10^−12^	0.15
2-keto-L-gluconic acid	Organic acid	7 × 10^−6^	0.18
benzoic acid	Organic acid	5 × 10^−13^	0.23
*L*-leucine	Amino acid	2 × 10^−6^	0.24
*L*-valine	Amino acid	7 × 10^−8^	0.26
galactinol	Sugar	3 × 10^−10^	0.29
*L*-threonic acid	Organic acid	1 × 10^−8^	0.34
*L*-threonine	Amino acid	6 × 10^−4^	0.36
*L*-isoleucine	Amino acid	4 × 10^−6^	0.36
β-sitosterol trimethylsilyl ether	Sterol and lipid	3 × 10^−2^	0.36
4-O-β-galactopyranosyl-D-mannopyranose	Sugar	2 × 10^−8^	0.38
*trans*-9-octadecenoic acid	Fatty acid	2 × 10^−5^	0.39
glyceryl-glycoside TMS ether	Sugar	4 × 10^−12^	0.40
glutamic acid	Amino acid	4 × 10^−6^	0.42
*L*-(-)-fucose	Sugar	7 × 10^−8^	0.45
glycine	Amino acid	1 × 10^−2^	0.48
9,12-octadecadienoic acid (Z,Z)	Fatty acid	1 × 10^−7^	0.50
2-piperidinecarboxylic acid	Organic acid	3 × 10^−2^	0.51
*D*-mannopyranose	Sugar	4 × 10^−9^	0.52
phosphoric acid	Organic acid	4 × 10^−8^	0.54
phenylalanine	Amino acid	2 × 10^−2^	0.55
*d*-galactose	Sugar	1 × 10^−7^	0.57
hexadecenoic acid	Fatty acid	8 × 10^−7^	0.58
*D*-sorbitol	Sugar	6 × 10^−5^	0.59
serine	Amino acid	1 × 10^−3^	0.60
*L*-aspartic acid	Amino acid	3 × 10^−5^	0.62
gulose	Sugar	1 × 10^−7^	0.63
*L*-proline	Amino acid	6 × 10^−5^	0.63
tyrosine	Amino acid	5 × 10^−4^	0.64
3-α-mannobiose	Sugar	2 × 10^−5^	0.68
*Myo*-inositol	Sugar	1 × 10^−6^	0.71
propanedioic acid	Organic acid	6 × 10^−5^	0.72
octadecanoic acid	Fatty acid	1 × 10^−3^	0.75
1-cyclohexene-1-carboxylic acid	Organic acid	1 × 10^−4^	0.81
pentitol	Sugar	5 × 10^−3^	0.90
*D*-mannitol	Sugar	3 × 10^−2^	0.92
*D*-glucose	Sugar	5 × 10^−3^	1.20
xylulose	Sugar	5 × 10^−4^	1.21
*D*-arabinose	Sugar	1 × 10^−5^	1.29
*D*-fructose	Sugar	1 × 10^−6^	1.29
*D*-erythro-pentitol	Sugar	7 × 10^−7^	1.41
2(3H)-furanone	Sugar	2 × 10^−4^	1.43
2-butenedioic acid	Organic acid	1 × 10^−5^	1.49
hexopyranose	Sugar	3 × 10^−2^	1.77
propanoic acid	Organic acid	2 × 10^−8^	2.43
*D*-gluconic acid	Organic acid	4 × 10^−8^	4.15
citric acid	Organic acid	7 × 10^−11^	4.80
urea	Organic acid	5 × 10^−3^	7.70
*N*-acetyl glucosamine	Amide	2 × 10^−11^	13.12
butanoic acid	Organic acid	8 × 10^−5^	13.56
sorbic acid	Organic acid	7 × 10^−9^	14.81
*D*-(+)-cellobiose	Sugar	3 × 10^−3^	15.15
3-hydroxyanthranilic acid	Organic acid	4 × 10^−10^	17.69
*D*-pinitol	Sugar	3 × 10^−14^	37.28

AF: artificial diet group; MF: mulberry leaf group. P: *p*-value. AF/MF: the mean value of AF/the mean value of MF. Data were expressed as mean ± SEM. Differences of data was assessed by Student’s *t*-test (Two tailed, Two-sample equal variance hypothesis). MF, *n* = 6 replicates; AF, *n* = 6 replicates.

## Supplementary Materials

The following are available online at https://www.mdpi.com/2075-4450/11/12/851/s1, Table S1: Metabolites identified in fecal of silkworm, Bombyx mori, by GC-MS. Table S2. Chemical components identified in mulberry leaf and artificial diet, by GC-MS. Table S3. Correlation analysis of metabolites identified in fecal of silkworm, *Bombyx mori*, by GC-MS. Figure S1: Relative Hierarchical cluster analysis and the heatmap of the identified metabolites in feces from different diets, Figure S2: Chemical structures of 13 amino acids and six lipids identified in feces from different diets, Figure S3: Chemical structures of sugars, organic acids and urea in feces from different diets.
