# Supplementary material for: Comparative Fecal Metabolomes of Silkworms Being Fed Mulberry Leaf and Artificial Diet
Source: Insects. 2020 Nov 30;11(12):851. doi: 10.3390/insects11120851 (PMC7759890; doi:10.3390/insects11120851)
Supplement: Supplementary file 1 [file insects-11-00851-s001.zip › supplementary.docx]

Supplementary materials

Comparative fecal metabolomes of silkworms being fed mulberry leaf and artificial diet

Dao-Yuan Qin ^1,2,3^, Gen-Hong Wang ^1,2,3^, Zhao-Ming Dong ^1,2,3^, Qing-You Xia ^1,2,3^, Ping Zhao ^1,2,3*^

^1^ State Key Laboratory of Silkworm Genome Biology, Southwest University, Chongqing, 400715, P. R. China; qdyddinsist@email.swu.edu.cn (Q.-D.Y.); [wanggh168@swu.edu.cn](mailto:wanggh168@swu.edu.cn) (W.-G.H.); [dongzhaoming@swu.edu.cn](mailto:dongzhaoming@swu.edu.cn) (D.-Z.M.); [xiaqy@swu.edu.cn](mailto:xiaqy@swu.edu.cn) (X.-Q.Y.)

2 Biological Science Research Center, Southwest University, Chongqing, 400715, China;

^3^ Chongqing Key Laboratory of Sericultural Science, Chongqing Engineering and Technology Research Center for Novel Silk Materials, Southwest University, Chongqing, 400715, China

***** Corresponding: [zhaop@swu.edu.cn](mailto:zhaop@swu.edu.cn) (Z.P); Tel.: +86-023-68250885;


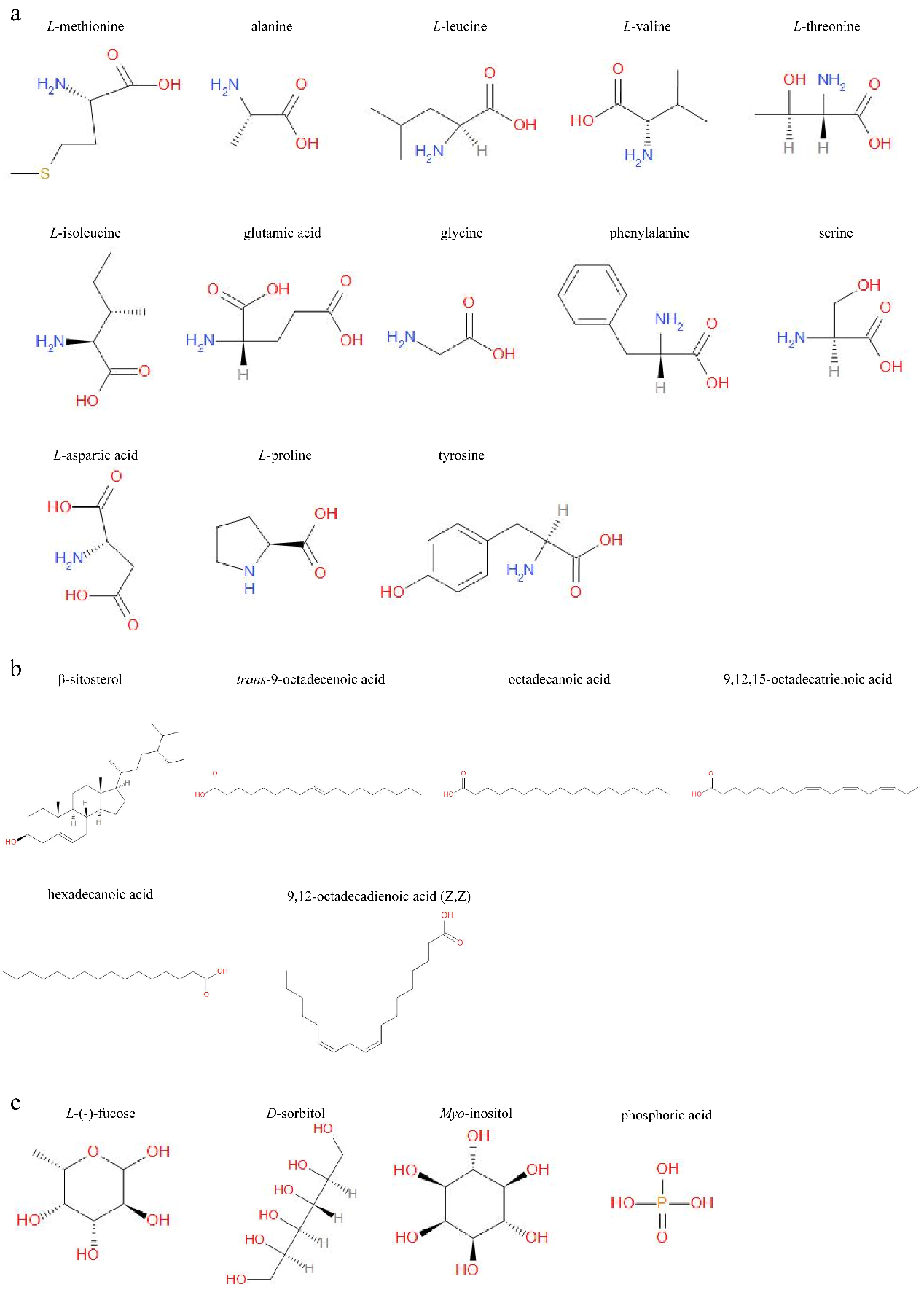


**Figure S1.** Chemical structures of 13 amino acids and six lipids identified in feces from different diets.


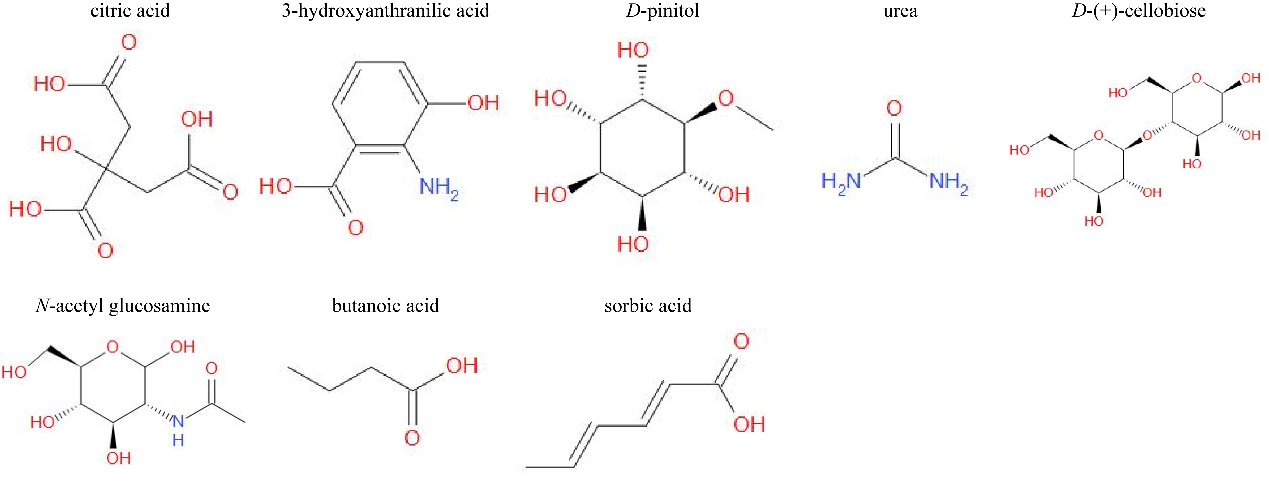


**Figure S2.** Chemical structures of sugars, organic acids, and urea in feces from different diets.


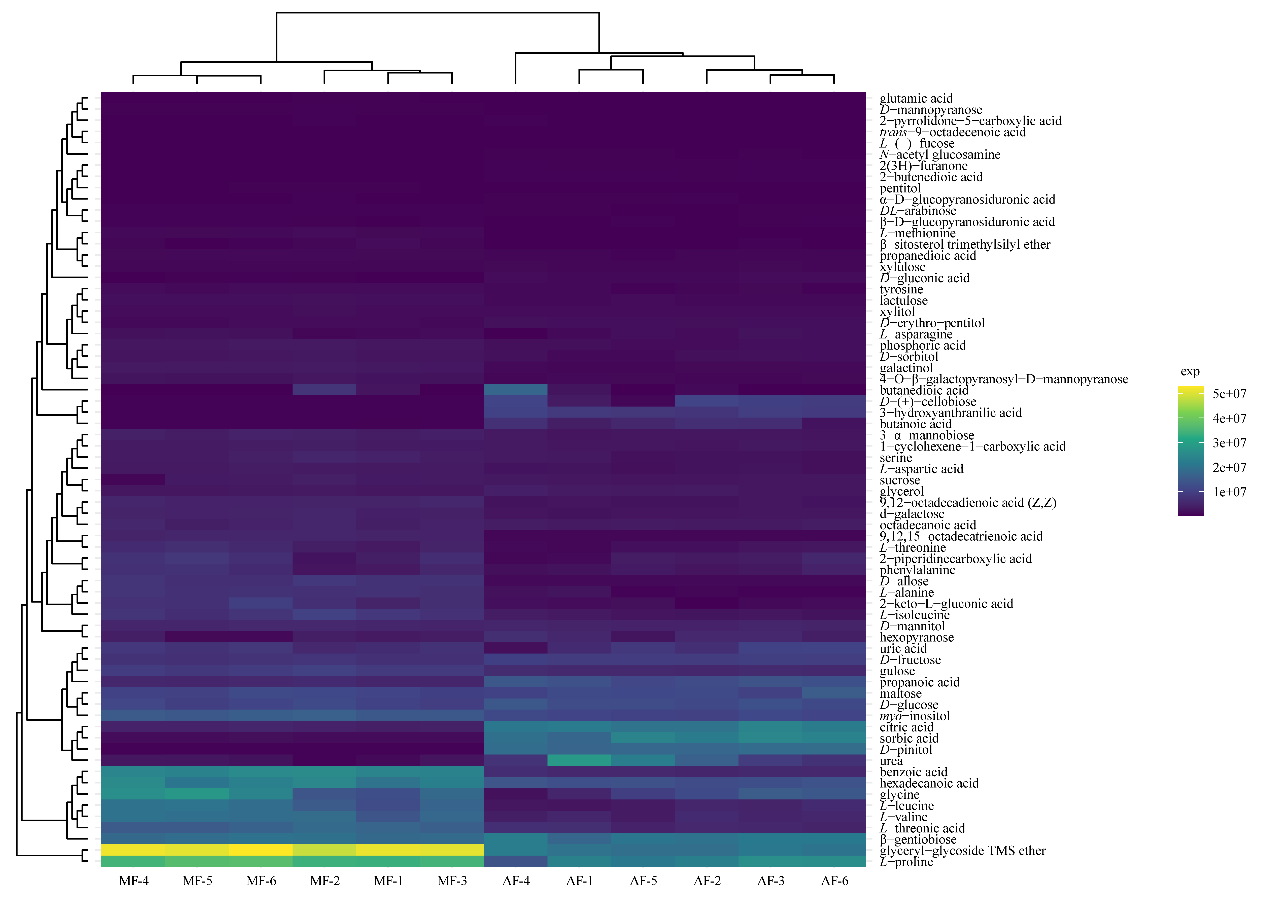


**Figure S3.** Relative Hierarchical cluster analysis and the heatmap of the identified metabolites in feces from different diets. MF and AF are larvae reared on fresh mulberry leaf and artificial diet, respectively. MF, n=6 replicates; AF, n=6 replicates.
